# Recurrent Ipsilateral C5 Nerve Palsy Associated With Hereditary Neuropathy With Liability to Pressure Palsy

**DOI:** 10.7759/cureus.55948

**Published:** 2024-03-11

**Authors:** Kei Nozue, Naoto Sugeno, Shun Ishiyama, Mikihiro Yoshida, Masashi Aoki

**Affiliations:** 1 Department of Neurology, Tohoku University Graduate School of Medicine, Sendai, JPN

**Keywords:** sleep habit, c5 nerve root, deltoid, pmp22, hnpp

## Abstract

Hereditary neuropathy with liability to pressure palsy (HNPP) is an autosomal dominant disorder caused by heteroplasmic deletion of the peripheral myelin protein 22 (PMP22) gene. HNPP typically presents with clinical features such as peroneal nerve palsy or cubital tunnel syndrome, which are caused by mechanical compression. Diagnosing cases where neuropathy is absent at the pressure site can be challenging. This is a case study of an 18-year-old man who underwent surgery on the left side of his neck over 10 years ago to remove lymphadenopathy. Following the surgery, he experienced recurrent weakness but only sought medical attention when muscle weakness persisted for longer than a week postoperatively. Upon admission, the patient exhibited neurological symptoms consistent with C5 neuropathy, mainly affecting the deltoid muscles. No serological abnormalities were found to be associated with neuropathy. Neither magnetic resonance imaging nor computed tomography scans detected any lesions around the C5 nerve root. The posture during sleep was believed to cause excessive extension of the C5 nerve root, leading to the assumption that there was some vulnerability in the nerve. A transient sensory loss in the area innervated by the ulnar nerve prompted us to examine the fluorescence in situ hybridization study on the blood sample, which revealed a deletion of the PMP22 gene. The patient was diagnosed with HNPP and was advised to avoid risky postures. Following the implementation of these lifestyle changes, he did not experience any further weakness in his shoulders.

## Introduction

Hereditary neuropathy with liability to pressure palsy (HNPP) is an autosomal dominant disorder associated with heteroplasmic deletion of the peripheral myelin protein 22 (PMP22) gene [1]. Most of the HNPP patients carry a 1.5 Mb deletion in chromosome 17p11.2-12, and a small number of HNPP patients carry PMP22 point mutations which result in premature stop codon [2]. These PMP22 gene abnormalities reduce PMP22 function to maintain the neuronal sheath, making peripheral nerves vulnerable to mechanical stimuli. Therefore, neuropathic symptoms of HNPP are usually preceded by trauma or physical exercise [3]. Because of these specifications, good examples of initial manifestations of HNPP are limited in peroneal, ulnar, and median nerves that have compression sites. Meanwhile, several atypical HNPP cases without episodes of nerve compression or abnormal compression sites can be found in the literature [4]. Current knowledge estimates the incidence of HNPP at 7-16 per 100,000 individuals [5], but these atypical cases may be overlooked and the actual number should be much higher. This report presents a case of HNPP with recurrent muscle weakness in an unusual part of the body, the shoulder area, particularly affecting the deltoid muscles, which ultimately resulted in the diagnosis of HNPP.

## Case presentation

**Figure 1:**
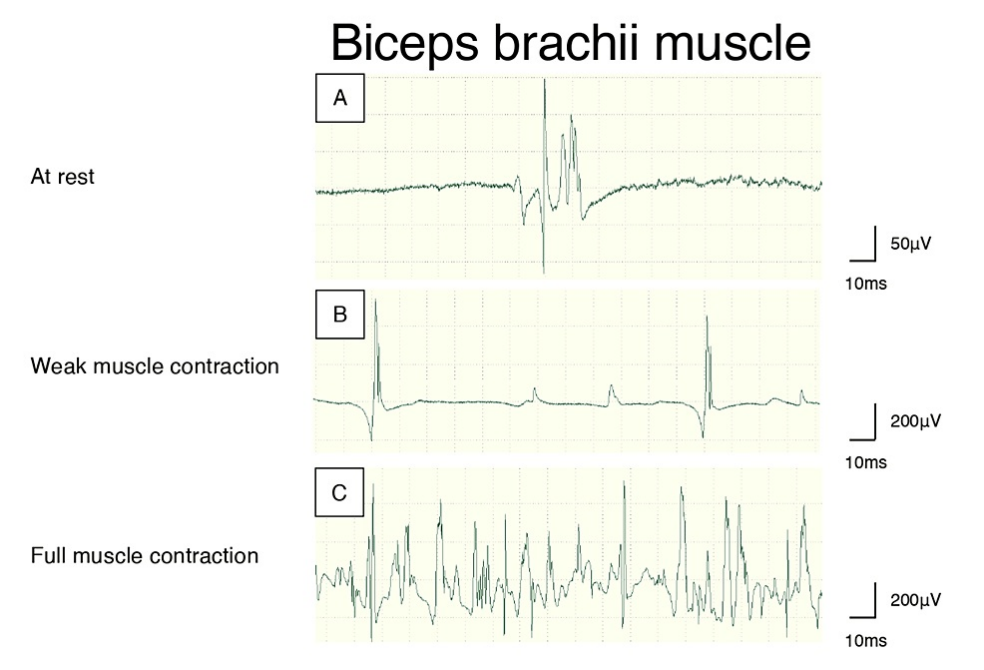
Needle electromyography findings. Examined in the left biceps brachii muscle. Fasciculation potentials are observed at rest (A). During voluntary movement, the recruitment is normal and interference is preserved (B and C).

This is a case of an 18-year-old Japanese man who suffered from purulent lymphadenitis and underwent a surgical procedure on the left side of his neck to remove lymphadenopathy when he was six years old. The operators checked cervical nerves like whether accessory nerves were preserved. Three months after the procedure, he woke up with numbness in his left arm and difficulty elevating it. These symptoms spontaneously alleviated within a week. Subsequently, he experienced similar episodes in his left arm every three months, which mostly resolved within a few weeks. He was admitted to our hospital due to the gradual worsening of his shoulder function after recovery. A neurological examination conducted soon after the recent attack showed proximal muscle weakness in the proximal site of his left upper limb and superficial sensory disturbance localized in the lateral side of his left upper arm (Table 1). The left biceps tendon and brachioradialis tendon reflexes were absent, while other reflexes were present. He did not exhibit any dysmorphic features, including pes cavus. He was hospitalized for further examination. The distribution of affected muscles and sensory problems was mostly consistent with left C5 neuropathy. However, cervical cord magnetic resonance imaging (MRI) did not reveal any lesions such as disc herniation, ossification of ligaments, inflammatory reactions, or tumors. Enhanced computed tomography of the whole body also failed to detect any abnormalities that could affect cervical nerves. The blood test, which included liver function, renal function, thyroid function, anti-GM1 IgG antibodies, and anti-nuclear antibodies, was normal. The cerebrospinal fluid was also normal and showed no elevation of protein or oligoclonal bands. The needle electromyography was examined from the proximal to distal sites of the upper and lower limbs. Neurogenic changes, such as fasciculation and fibrillation potentials, were only detected in the left biceps brachii (Figure 1). The nerve conduction study (NCS) did not indicate any signs of peripheral neuropathy.

**Table 1:** Distribution of affected muscles and innervating nerves. Manual muscle testing was used to estimate muscle strength, while computed tomography was used to evaluate muscle atrophy. When considered in peripheral nerves, it spans four nerves (axillary, suprascapular, long thoracic, and musculocutaneous nerves), but in nerve roots, it can be explained by C5 alone (bold font). MMT: Manual muscle testing.

	Muscle	Root	Trunk	Nerve	MMT	Atrophy
Right	Trapezius	XI, C1-4	-	accessory	5	-
	Deltoid	C5	superior	axillary	2	+
	Infraspinatus	C5-6	superior	suprascapullar	3	-
	Serratus Anterior	C5-6	-	long thoraic	4	-
	Biceps Brachialis	C5-6	superior	musculocutaneous	4	-
	Triceps	C7	middle	radial	4	-
	Brachioradialis	C6-7	superior	radial	5	-
	Extensor Carpi Radialis Longus	C6-7	superior	radial	5	-
	Extensor Carpi Ulnaris	C6-7	inferior	radial	5	-
	Extensor Digitorum Communis	C8	inferior	radial	5	-

**Figure 2:**
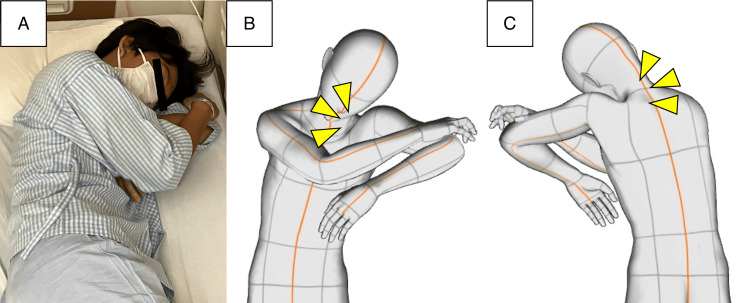
Sleeping posture in the current case. The patient habitually adopts a posture with the left shoulder joint in internal rotation and extension, and the head resting on the shoulder and upper arm (A). The posture is drawn three-dimensionally in Clip Studio Paint (CELSYS, Tokyo, Japan) (B and C).

As previously experienced, the strength of his left shoulder muscles gradually improved. However, upon waking, he noticed numbness and mild weakness on the lateral side of his left hand, mainly in the abductor digiti minimi. These symptoms corresponded to left ulnar nerve palsy, which disappeared immediately after four hours. As every symptom developed during sleep, we were concerned about his sleeping habits. The video footage captured at night showed the patient maintaining a fixed posture. He was lying on his left side with his left arm abducted and flexed, and his neck was flexed as well (Figure 2). These observations led us to investigate whether his sleeping habits were contributing to the development of recurrent neuropathy. However, entrapment C5 neuropathy is not a common occurrence, so we explored other factors that could be contributing to the patient's neuronal vulnerability to external stimuli. The fluorescence in situ hybridization analysis revealed that 97% of the leukocytes had a single PMP22 gene, leading to a diagnosis of HNPP. To prevent nerve damage, it is strongly recommended to avoid risky postures and to use a suitable pillow. After implementing these lifestyle changes, the individual reported no further weakness in his shoulders.

## Discussion

HNPP is an autosomal dominant disorder caused by the deletion of the PMP22 gene [1]. PMP22 protein is located in cholesterol-enriched membrane domains that are closely associated with the underlying actin network [6]. Reduced levels of PMP22 protein in neurons may cause instability of the myelin sheath, making it vulnerable to compression. HNPP typically affects the peroneal, median, and ulnar nerves due to entrapment at the fibula head, carpal tunnel, or cubital tunnel, respectively [7]. However, our case presented with repeated and almost exclusive C5 neuropathy. The C5 nerve is one of the origins of the brachial plexus. Although rare, some reports have documented cases of HNPP presenting with brachial plexopathy and shoulder abduction difficulties [8,9]. In our case, the main nerve involved was C5, but C7 and ulnar nerves also showed minimal involvement. Then it is possible that multiple neuropathies in our case were recognized as brachial plexopathy in our case [10]. However, unlike our case, all previous cases had some of the neuropathic features, such as absent Achilles tendon reflexes, or prolonged distal latencies on NCS. Therefore, the distribution of affected nerves in our case, which lacks features of polyneuropathy, differs significantly from previously known HNPP cases. Two potential explanations could account for the atypical presentation of our case. Firstly, hyperextension of the C5 nerve due to sleeping habits, and secondly, a history of lymph node dissection on the same side. The implemented lifestyle changes aimed at preventing symptom recurrence support the theory that nerve entrapment is consistent with the pathophysiology of HNPP. However, a clear link between the patient's surgical procedure and the neuropathy could not be established. There is also no clear family history. It is only possible to state that this unusual benign muscle weakness occurred after lymphadenectomy. It is speculated that some kind of elastic chord might be produced neighboring the C5 nerve due to the lymphadenectomy, but these structures are not detected by imaging analyses.

Currently, there is no specific treatment available for HNPP, such as PMP22 replacement. However, lifestyle changes may help prevent disease progression. Therefore, it is important to consider the possibility of HNPP in patients with compression neuropathy, even if the neuropathy was not generated in the usual compression site.

## Conclusions

HNPP is a disease closely linked to PMP22 deletion, while PMP22 duplication results in the Charcot-Marie-Tooth disease type 1A (CMT1A). HNPP can also develop in a polyneuritis-like form after a long course, which leads to reduced ADL. Unlike CMT1A, lifestyle modifications can prevent disease progression in HNPP. Therefore, HNPP should be actively suspected when the patient presents with recurrent muscle weakness and sensory deficits in a specific area, even when the site of lesion is suspected to be a nerve root.
